# The global implications of U.S. withdrawal from WHO and the USAID shutdown: challenges and strategic policy considerations

**DOI:** 10.3389/fpubh.2025.1589010

**Published:** 2025-06-02

**Authors:** Auwal Rabiu Auwal, Abubakar Sunusi Ishak, Shuaibu Saidu Musa, Abbas Musa, Abubakar Saadu, Anila Riaz

**Affiliations:** ^1^Department of Biochemistry, Faculty of Sciences, Chulalongkorn University, Bangkok, Thailand; ^2^School of Global Health, Faculty of Medicine, Chulalongkorn University, Bangkok, Thailand; ^3^Department of Nursing Sciences, Ahmadu Bello University, Zaria, Nigeria; ^4^Department of Biochemistry, Bayero University, Kano, Nigeria; ^5^Department of Biochemistry, Chulalongkorn University, Bangkok, Thailand

**Keywords:** USAID, WHO, pandemic preparedness, global health security, HIV/AIDS

## Abstract

The U.S. withdrawal from the WHO and the shutdown of USAID disrupt global health governance, threatening disease surveillance and reversing progress made in combating HIV/AIDS, tuberculosis, and malaria through initiatives like PEPFAR. The WHO's central role in coordinating global health responses, exemplified by its leadership in the COVAX initiative, is critical to pandemic preparedness and equitable vaccine distribution. The disengagement decision undermines humanitarian relief efforts, particularly in fragile states where U.S. contributions are vital for food security, education, and healthcare. This policy brief outlines actionable recommendations to mitigate the risks of U.S. disengagement, focusing on regional health capacity-building, public-private partnerships, and the continuation of critical investments in global health systems.

## 1 Introduction

The world faces complex, multifaceted health crises, with infectious diseases, antimicrobial resistance, climate-induced health challenges, and political instability becoming more interconnected. Amidst these global challenges, the United States' recent decision to withdraw from the World Health Organization (WHO) and shutter USAID operations marks a dramatic policy shift with significant consequences for international health governance. Historically, the U.S. has been a pillar in shaping the global health landscape, contributing substantially to the establishment of WHO and providing long-term financial support through USAID (1, 2).

The implications of this withdrawal are far-reaching. The U.S. has long been a key player in promoting global health equity, disease prevention, and emergency response. Stepping back risks creating substantial gaps in the global health system, particularly for vulnerable populations. The closure of USAID, which provides essential funding and expertise in countries battling HIV/AIDS, tuberculosis (TB), and malaria, is particularly concerning given its direct impact on life-saving health initiatives (3).

The withdrawal also threatens the effectiveness of global governance structures, as U.S. leadership has historically helped to shape multilateral responses to pandemics and health emergencies. With global health challenges becoming more complex and interconnected, the absence of U.S. support jeopardizes coordinated global efforts to tackle emerging diseases and maintain health security (4, 5).

This policy brief explores the global implications of U.S. disengagement, specifically focusing on the disruption of disease surveillance systems, humanitarian relief efforts, and undermining research initiatives. It also proposes strategic recommendations to address these challenges and ensure continued global health resilience and international cooperation.

## 2 Policy options and implications

### 2.1 Disruption of infectious disease control and surveillance

One of the most immediate consequences of U.S. withdrawal is the destabilization of global disease surveillance and response systems. USAID has long been a critical partner in the global fight against infectious diseases, including HIV/AIDS, tuberculosis (TB), and malaria. Through initiatives such as the President's Emergency Plan for AIDS Relief (PEPFAR), the U.S. has supported the treatment of over 18 million individuals living with HIV globally, contributing to a near-halving of AIDS-related deaths worldwide over the past decade (6). Furthermore, USAID's work in strengthening TB programs has been vital in achieving significant reductions in the disease's burden in high-incidence countries like India and South Africa (7, 8).

Similarly, the WHO's coordinating role in pandemic preparedness and response has been central to global health security. WHO's Global Outbreak Alert and Response Network (GOARN) provides rapid, real-time data analysis and coordinates medical missions during disease outbreaks. The COVID-19 pandemic underscored the importance of the WHO's role in global health, with the organization launching COVAX, which distributed over 2 billion vaccine doses to 144 countries, a cornerstone in the global effort to control the pandemic (9, 10).

However, WHO has faced its criticisms. Allegations of delayed emergency declarations, political influence, and data transparency concerns have emerged, particularly during the early stages of the COVID-19 crisis. These criticisms were not isolated to the U.S.; countries like Japan and Germany also voiced concerns regarding the WHO's delayed actions. Despite these issues, the WHO's role in coordinating global health responses cannot be underestimated. The withdrawal of the U.S. from WHO diminishes the opportunity for reform and compromises the organization's ability to adapt and respond to emerging global health threats (11).

### 2.2 Humanitarian and developmental setbacks

Beyond health, USAID has been instrumental in providing humanitarian relief and promoting sustainable development. USAID's Feed the Future initiative has lifted over 23 million people out of extreme poverty, while its nutrition programs have improved the lives of more than 12 million children globally. The agency's efforts have been particularly impactful in fragile states such as Yemen and South Sudan, where USAID has played a pivotal role in providing food security, education, and health services (12, 13).

During the West African Ebola outbreak in 2014, USAID mobilized over $2.4 billion to support epidemic control efforts, including building treatment centers and supporting local health systems. The U.S. government's swift response to the Ebola crisis is an example of the essential role USAID has played in mitigating the effects of global health emergencies (14, 15).

The cessation of these programs poses significant risks to global stability. Without USAID's contributions, humanitarian organizations may struggle to fill the void left by the U.S. leading to increased political instability, poverty, and disease in vulnerable regions (16).

### 2.3 Geopolitical consequences

The U.S. withdrawal from WHO and USAID also carries profound geopolitical implications. Development aid has long been a key tool of U.S. soft power, strengthening diplomatic relations and promoting global stability. However, the U.S.'s retrenchment from these platforms may create a power vacuum that rival nations are eager to fill (17).

For example, China's Belt and Road Initiative (BRI), which includes health infrastructure investments, has been expanding its influence in countries previously reliant on U.S. aid (18). China's health diplomacy, including vaccine donations and medical missions, contrasts sharply with the transparency and rights-based approaches championed by USAID. The absence of U.S. leadership may weaken global norms around health equity and human rights, potentially enabling authoritarian regimes to impose their models of governance in development projects (18).

This shift in global leadership could lead to fragmented health responses, undermining efforts to address cross-border health threats like antimicrobial resistance, pandemics, and climate-related health crises.

### 2.4 Consequences for research and innovation

Scientific collaboration has been another cornerstone of U.S. global health leadership. Through initiatives like PEPFAR, the National Institutes of Health (NIH), and the CDC, the U.S. has funded groundbreaking research in vaccine development, genomics, and digital health technologies. USAID's Center for Innovation and Impact (CII) has been particularly instrumental in advancing scalable health technologies, including mobile health platforms and AI-based diagnostics (19).

Reducing U.S. support for global health research would hinder scientific innovation and undermine collaborative research efforts between low- and high-income countries. This erosion of scientific partnerships could slow progress on critical issues such as pandemic preparedness, vaccine development, and the fight against antimicrobial resistance (20).

## 3 Actionable recommendations

Several strategic, well-coordinated interventions must be implemented to mitigate the adverse effects of the U.S. withdrawal from WHO and the shutdown of USAID operations. These recommendations aim to safeguard global health security, maintain humanitarian support, and foster resilience within the international health governance framework.

### 3.1 Implement a phased and coordinated transition strategy

A structured phased withdrawal is essential to minimize the disruptive impact of U.S. disengagement. The U.S. government should:

- Allocate bridging grants to support critical ongoing health initiatives, particularly in HIV/AIDS treatment, TB control, maternal and child health, and nutrition programs. This funding will help smooth the transition by keeping essential programs running while new funding mechanisms are secured.
- Establish collaborative transition task forces involving USAID, WHO, and local stakeholders. These task forces should design tailored transition plans that prioritize knowledge transfer, capacity building, and stakeholder alignment, ensuring that regional partners can assume leadership roles.
- Ensure the continuation of multi-year commitments, particularly in emergency health response, immunization campaigns, and epidemic control. These programs often span several years, and premature discontinuation can have devastating consequences.
- Create multi-donor trust funds that pool resources from a wide range of global partners to sustain funding for high-impact programs. These funds should be transparent and have clear reporting mechanisms to guarantee accountability.
- Third-party performance audits must be conducted regularly during the transition phase to evaluate the program handovers' effectiveness and maintain the integrity of ongoing operations.

By adopting this structured approach, the U.S. can ensure that health and development programs do not collapse after its withdrawal. Moreover, this will help build trust with other international donors and ensure a smooth shift to regional leadership.

### 3.2 Strengthen regional health institutions and technical capacity

Given the global health risks posed by U.S. disengagement, the need for strong, independent regional health institutions has never been greater. The U.S. should:

- Provide targeted financial and technical support to regional organizations like Africa CDC, the ASEAN Health Cluster, and the Pan American Health Organization (PAHO). These bodies should be empowered to lead on health security issues like disease surveillance, emergency response, and vaccine distribution.
- Fund capacity-building initiatives that enhance laboratory infrastructure, surveillance systems, and epidemiological modeling. This will increase the regions' ability to swiftly and effectively identify, monitor, and respond to emerging diseases.
- Support regional vaccine production hubs, such as the Institut Pasteur de Dakar in Senegal, to reduce dependency on global supply chains and ensure equitable access to vaccines, particularly for low- and middle-income countries (LMICs). This investment is essential in the fight against diseases like COVID-19, malaria, and TB, where access to vaccines and treatments remains uneven.
- Facilitate inter-regional cooperation on health research and innovation, particularly in infectious disease surveillance, medical diagnostics, and low-cost treatment development.

By strengthening regional institutions and health infrastructures, the U.S. can help ensure that the global health system remains resilient despite geopolitical and financial challenges. Empowering regional leadership in health can also prevent larger powers from monopolizing health responses and ensure that solutions stay equitable and locally relevant.

### 3.3 Leverage public-private partnerships for innovation and funding

The future of global health lies in the collaboration between governments, the private sector, and civil society organizations. To fill the funding gap left by the U.S. withdrawal, the U.S. should:

- Facilitate strategic public-private partnerships (PPPs) that leverage the strengths of both sectors. For example, the U.S. can work with global health organizations, pharmaceutical companies, and local governments to scale up the development and distribution of affordable vaccines, diagnostic tools, and treatments.
- Invest in technological innovations that can transform health delivery in LMICs, such as mobile health platforms, AI-driven diagnostics, and telemedicine solutions. This will allow for more cost-effective healthcare delivery in resource-limited settings.
- Development Impact Bonds (DIBs) should be innovative financing mechanisms. Investors provide upfront capital to fund health programs in exchange for repayment based on achieving measurable health outcomes. This approach aligns private investment with public health goals and efficiently allocates resources.
- Promote the local manufacturing of health technologies through partnerships with local businesses. This will reduce dependence on international supply chains and improve self-reliance in health systems.

By expanding PPPs, the U.S. can help stimulate local innovation, provide sustainable financing for health programs, and ensure that global health solutions are both scalable and accessible to the communities that need them most.

### 3.4 Continue investing in global health security and public goods

Despite the reduced U.S. role, maintaining strategic investments in global health security remains paramount. The U.S. should:

- Reaffirm its commitment to global health security initiatives, including Gavi, the Global Fund, and CEPI. These organizations have been instrumental in global vaccination efforts, and continued U.S. support is vital to maintaining the momentum of global health programs.
- Invest in pandemic preparedness by supporting the International Health Regulations (IHR) and investing in global disease surveillance systems. Strengthening these systems ensures that the world remains better prepared for the next global health crisis.
- Support global research efforts on issues like antimicrobial resistance, zoonotic diseases, and climate-related health impacts, which will require collective action to address effectively. These investments will help safeguard global health security and maintain U.S. influence in shaping international health norms.
- Engage in bilateral agreements with key health partners to address specific health challenges in vulnerable regions, ensuring that critical initiatives continue even if U.S. involvement in multilateral platforms is reduced.

These sustained investments will ensure that global health systems remain robust, resilient, and ready to respond to emerging health threats. Continued U.S. participation will be essential in maintaining a coordinated and effective international response to health crises.

### 3.5 Promote inclusive and transparent global health governance

Rather than withdrawing from global health platforms, the U.S. should advocate for reforming institutions like WHO to ensure they are more inclusive and effective. The U.S. should:

- Advocate for greater representation of low and middle-income countries (LMICs) in decision-making processes within WHO and other global health organizations. This will ensure that health solutions are tailored to the needs of the most vulnerable populations.
- Push for greater transparency and accountability in global health governance by supporting independent oversight bodies like the Independent Oversight and Advisory Committee (IOAC), which evaluates the performance of WHO and other international health initiatives.
- Support South-South cooperation, encouraging knowledge-sharing and collaboration between countries facing similar health challenges. This will foster peer learning and enhance the effectiveness of health interventions in LMICs.
- Reform global health financing mechanisms to ensure that funding is allocated more equitably and transparently, prioritizing health programs in countries most at risk of emerging diseases.

By advocating for inclusive governance, the U.S. can ensure that global health systems remain responsive to the needs of all countries, particularly those most vulnerable to health crises.

## 4 Conclusion

The U.S. withdrawal from WHO and USAID operations represents a monumental shift in the global health landscape. While domestic political considerations may drive this disengagement, the consequences for international health security, humanitarian aid, and scientific collaboration are profound. The U.S. has been a cornerstone of the global health system, and its absence risks destabilizing critical health programs, weakening multilateral health responses, and undermining the progress made in addressing global health inequities.

However, the U.S. can play a constructive and influential role in global health, even in a reduced capacity. By adopting a phased withdrawal strategy, supporting regional institutions, leveraging public-private partnerships, and continuing to invest in global health security, the U.S. can help ensure that the global health system remains resilient and capable of responding to emerging health challenges.

Ultimately, the U.S. must recognize that global health is an interconnected system. Its disengagement will weaken international health responses and diminish its leadership role in shaping the future of global health governance. A reimagined engagement strategy that balances national priorities with global responsibilities will ensure global health systems' continued resilience, equity, and effectiveness.

## References

[1] Gostin LO Friedman EA Wetter S. Dismantling US Global Health Aid. Health Affairs Forefront. (2025). 10.1377/forefront.20250213.2188437562419

[2] Mildenberger M. Trump and Musk's War on USAID Will Cost Lives. International Policy Digest. (2025). Available online at: https://intpolicydigest.org/trump-and-musk-s-war-on-usaid-will-cost-lives/

[3] World Health Organization. WHO Delivering on Its Commitment to Protect and Improve People's Health: Stories of Healthier Populations, Access to Services and Emergency Response. World Health Organization (2024). Available online at: https://www.who.int/publications/i/item/9789240104884

[4] Liu Y Hall BJ Ren M. How the US presidential election impacts global health: governance, funding, and beyond. Global Health Res Policy. (2024) 9:49. 10.1186/s41256-024-00391-w 39563373 PMC11577883

[5] Kickbusch I. US exit from WHO: it is about much more than WHO. Lancet. (2025) 405:444–6. 10.1016/S0140-6736(25)00163-1 39884296

[6] *Global AIDS Update* (2023). Available online at: https://www.unaids.org (accessed April 19, 2025).

[7] Ogieuhi IJ Ajekiigbe VO Aremu SO Okpujie V Bassey PU Babalola AE. Global partnerships in combating tropical diseases: assessing the impact of a US withdrawal from the WHO. Trop Med Health. (2025) 53:36. 10.1186/s41182-025-00722-8 40065473 PMC11892267

[8] Burki T. The fate of tuberculosis programmes without USAID. Lancet Microbe. (2025) 101145. 10.1016/j.lanmic.2025.101145. [Epub ahead of print]. 40220769

[9] *COVAX Reaches Over 2 Billion Vaccines Delivered* (2022). Available online at: https://www.who.int (accessed April 19, 2025).

[10] Piwowar-Sulej K Malik S Shobande OA Singh S Dagar V. A contribution to sustainable human resource development in the era of the COVID-19 pandemic. J Bus Ethics. (2024) 191:337–55. 10.1007/s10551-023-05456-3 37359809 PMC10240448

[11] McCarthy M Murphy K Sargeant E Williamson H. Policing COVID-19 physical distancing measures: managing defiance and fostering compliance among individuals least likely to comply. Polic Soc. (2021) 31:601–20. 10.1080/10439463.2020.1869235

[12] Yemen Tigray and Global Health Impact Reports (2023). Available online at: https://www.usaid.gov (accessed April 19, 2025).

[13] Doggett D. Analyzing the Impacts of International versus Community Based Organizations on Food Security. University of Michigan Library (2024). 10.7302/22668

[14] Dahl BA. CDC's response to the 2014–2016 Ebola epidemic—Guinea, Liberia, and Sierra Leone. MMWR Suppl. (2016) 65:12–20. 10.15585/mmwr.su6503a3 27388930

[15] Holmberg M. On Viral Terrain: The Shifting Imaginaries of Geography and Infectious Disease Risk in USAID's PREDICT. Vancouver: University of British Columbia (2024).

[16] Kostin M Korotayev A. USAID democracy promotion as a possible predictor of revolutionary destabilization. Compar Sociol. (2024) 23:240–78. 10.1163/15691330-bja10102

[17] Haug S Novoselova A Klingebiel S. Trump's assault on foreign aid: Implications for international development cooperation. In: IDOS Discussion Paper (2025). Report No.: 3960212488.

[18] Youde J. Global health governance in international society. Glob Gov. (2017) 23:583–600. 10.1163/19426720-02304005

[19] Reid MJ Bunnell R Dokubo EK Nkengasong J. Programme science in PEPFAR: a pathway to a sustainable HIV response. J Int AIDS Soc. (2024) 27:e26244. 10.1002/jia2.26244 38982891 PMC11233845

[20] Yazdi-Feyzabadi V Haghdoost A-A McKee M Takian A Bradley E Brugha R. The United States withdrawal from the world health organization: implications and challenges. Int J Health Policy Manag. (2025) 14:1–4. 10.34172/ijhpm.9086 PMC12089832 40767200

